# Effects of steam-flaked grains on foals’ growth and faecal microbiota

**DOI:** 10.1186/s12917-021-02994-8

**Published:** 2021-09-04

**Authors:** Xiao Bin Li, Xin Xin Huang, Chang Jiang Zang, Chen Ma, Kai Xu Chen, Guo Dong Zhao, Qian Li, Xuan Yue Li, Wen Jie Zhang, Kai Lun Yang

**Affiliations:** College of Animal Science, Xinjiang Key Laboratory of Herbivore Nutrition for Meat & Milk Production, Xinjiang Agricultural University, Xinjiang 830052 Urumqi, China

**Keywords:** Foal, Steam-flaked grains, Corn, Oat, Barley, Growth performance, Faecal microbiota

## Abstract

**Background:**

There is little objective information concerning the effect of steam-flaked grains on foal’s growth performance and faecal microbiota. To determine the effects of steam-flaked grains on foal’s growth performance and faecal microbiota, faecal samples were collection from 18 foals which had been fed either corn, oat or barley diets over the 60 days of the experiment. Body weight and conformation measurements were collected. Next-generation sequencing of the V3 + V4 region of the 16 S rRNA gene was used to assess the microbial composition of faeces. Alpha diversity, Venn graph, Relative abundance and beta diversity are presented.

**Results:**

There was a significantly higher larger increase in the body weight of those foals fed barley compared to either corn or oats. There were also significant changes in the Alpha diversity of the gut microbiota. The Shannon and Simpson indices were significantly higher in the barley fed group than those fed corn or oats. The Chao1 index was significantly higher in the oat fed group than the corn or barley fed groups. There were significant changes in the relative abundance of bacteria in the microbiota in terms of phylum, family and genus. The histogram of LDA value distribution showed that the 12 statistically different biomarkers of the bacteria were present. Tax4Fun function annotation clustering heat map showed that functional information was detected from 26 species of bacteria in faecal samples from the foals.

**Conclusions:**

Differences by starch sources were found in overall growth of the foals and in the faecal microbiota if either supplementary corn, oat or barley was fed. Further studies are required to determine the potential impact of the changes in the microbiota on the health and development of foals fed cereal starch of different sources.

## Introduction

The gut microbiota performs essential roles in the maintenance of growth and health of animals [1]. In the equine species, the gut bacterial microbiota is essential due to its role in for cellulose fermentation and short-chain fatty acid production which are the primary energy sources for growth and health [2]. Equines are hind-gut fermenters [3] and thrive on high-fibre, low-energy fodder due to the complex microbial community in their gut. Gut microbiota composition is not constant, but may change over time due to factors such as variation in diet [4], age, management, gut disease [5] and weaning. Cereal grains such as corn, oat, and barley are often included in equine diets [6] to increase energy uptake [7]. All three cereal species consist of amylose and amylopectin, but differ in the proportion of those polysaccharides, and also in the morphology of the starch granule [8, 9]. In the small intestine, the starch of grains is digested by amylolytic enzymes and absorbed as glucose. Starch structures which are not completely digested in the small intestine will be delivered to the caecum and colon, and become fermented by local bacterial microbiota. In the hindgut, starch fermentation can lead to increased numbers of amylolytic bacteria, including lactobacilli and streptococci. This can increase the volatile fatty acid and lactic acid concentrations, decrease pH, and decrease the number of cellulolytic bacteria [10–12]. To improve the efficiency of grain feeding and to prevent diseases associated with the hind gut fermentation of starch, the digestibility in the small intestine must be enhanced. Currently, this is mainly done by the choice and processing of the appropriate cereal grains. Processing changes the physical and chemical structure [8, 13]. Processing methods that utilize a combination of heat, moisture and pressure disrupt the starch granule structure, destroy crystalline starch formations, increase the water solubility which all enable physically exposure amylolytic enzymes [8, 14]. There are no studies on how the source of the starch and its processing affect the growth of foals and the dynamics of the hindgut microbiota. We hypothesized that changes in the hindgut bacterial community is a response to dietary starch and the character of change is affected by the processing and source of starch. The objective was to compare the effects on equine hindgut microbiota and growth when adding processed oats, corn or wheat to a forage-based diet.

## Materials and methods

### Animals

In our study, We chose the Kazakh Horse, The Kazhorse is a species of local breed in China [15]. Eighteen healthy 5-month-old weaned foals of the Kazakh toad breed with a starting bodyweight of 112.4 ± 7.5 kg was included in the study. The foals were born in March, weaned in August, and the trial ran between August and October(60 days). Foals were selected from herds occupying the same local pasture and were clinically normal at inspection and had no history of systemic illness. Before weaning the foals had been dewormed with Ivermectin.The foals were randomly allocated to one of the 3 diets groups: corn group, oat group, or barley group. Each group contained 6 foals.

### Feed

All foals were fed alfalfa and hay and a basic concentrate supplement which was completed with either steam-pressed corn, oats or barley depending on the foal’s diet group allocation (see Table 1 for quantity of supplement fed). The alfalfa and hay were mixed evenly at a ratio of 2:1, and be able to consume 1.5 kg of dry matter (DM) for each 100 kg of body weight.The amounts of starch fed were set at 2 g starch (DM) /kg bodyweight [6]. With a mean foal weight between birth and 30 days of 112 kg the required amount of additional starch was 224 g/ day. The average weight from 31 to 60 days of age was 126 kg and the starch addition was set at 252 g (Tables 1 and 2), Within 10 days of the pre-feeding period, let the foals gradually adapted to the adjustment of dietary starch. The nutrient levels of the foal’s diet during the test period were shown in Table 3.

**Table 1:** Supplementary feeding amount of concentrate supplement

Groups	0–30 d(g)	31–60 d(g)
Corn	650	710
Oat	660	720
Barley	660	720

**Table 2:** Composition of concentrate supplement(%)

The raw material	Corn group	Oat group	Barley group
Steam-pressed corn	60	--	--
Steam-pressed oats	--	66	--
Steam-pressed barley	--	--	66
Soybean meal	36	30	30
Calcium hydrogen phosphate	2	2	2
Limestone	1	1	1
Premix^a^	0.5	0.5	0.5
Salt	0.5	0.5	0.5

^a^The premix provided the following per kg of concentrate supplement: Vitamin A 3000 IU,Vitamin B_1_ 20 mg,Vitamin B_2_ 20 mg,Vitamin B_6_ 6 mg,Vitamin C 20 mg,Vitamin D 1000 IU,Vitamin E 500 IU, Pantothenic acid 10 mg, Nicotinamide 100 mg, Cu 25 mg, Fe 107 mg, Mn 81 mg, Zn 74 mg, I 6 mg, Se 14 mg,Co3 mg, Choline chloride 120 mg

**Table 3:** Nutrient levels of hay, alfalfa and concentrate Supplements (Dry Matter,DM basis)

Nutrient	Hay	Alfalfa	Corn group	Oat group	Barley group
Dry Matter,DM (%)	89.75	87.56	92.44	93.28	91.93
Organic Matter,OM (%)	91.80	92.21	94.46	93.32	93.90
Crude Protein,CP (%)	10.75	20.56	26.26	26.09	26.86
Gross Energy,GE (MJ/kg)	18.93	19.53	18.25	18.29	18.26
Starch (%)	3.63	4.46	37.68	37.00	38.27
Neutral Detergent Fiber,NDF (%)	60.00	52.60	24.46	27.04	24.79
Acid Detergent Fiber,ADF (%)	41.82	45.21	5.10	9.09	5.71
Calcium,Ca (%)	0.33	1.50	1.06	1.45	1.14
Phosphorus,P (%)	0.27	0.25	0.94	1.02	1.02

### Management

During the 60 days study, the foals were individually housed in partially covered confinements of red brick (8 × 6 m). Each unit contained an automatic water source and the feeding area was equipped with red brick floor and a large wooden tub secured to the wall. Horses were turned out each day in dry lot paddocks after feeding.

### Weight and body measure data collection

The body weight was assessed using portable scales ( Loadometer is produced by Tru-Test Company in New Zealand, model: ID3000, accuracy 0.5 kg, measuring range 0.0-1000 kg. The product was purchased from Beijing Dongfang Lianming Technology Development Co., Ltd., China ), the height at the withers body and length from the point of the shoulder to the ischial tuberosity were determined with a measuring rod, and the chest and cannon (metacarpal) circumference were determined with a tape measure (range 5 m). The data were collected before the first feeding in the morning on the first, thirtieth, and sixtieth day of treatment.

### Faecal sample collection

Faecal samples were collected by rectal palpation using one rectal sleeve per foal per sampling. Samples were stored in plastic sterile containers (RNA free) and snap frozen in liquid nitrogen before storage at -70℃ until DNA extraction. Samples were collected before the first feed in the morning on the sixtieth day of treatment.

### DNA extraction

Total genome DNA from samples was extracted using a CTAB/SDS method [16]. DNA concentration and purity was assessed on 1 % agarose gels. DNA was diluted to 1 ng/µl using sterile water.

### 16 s rRNA gene amplification and sequencing

PCR amplification of the V3 + V4 region of 16 S rRNA gene was undertaken. The primers used for the amplification were 341 F: CCTAYGGGRBGCASCAG and 806R: GGACTACNNGGGTATCTAAT.

16 S rRNA genes were amplified using the specific primer with the barcode. All PCR reactions were carried out in 30µL reactions with 15 µL of Phusion® High-Fidelity PCR Master Mix (New England Biolabs); 0.2 µM of forward and reverse primers, and approximately 10 ng template DNA. Thermal cycling consisted of initial denaturation at 98℃ for 1 min, followed by 30 cycles of denaturation at 98℃ for 10 s, annealing at 50℃ for 30 s, and elongation at 72℃ for 30 s. Finally 72℃ for 5 min [16–18].

Sequencing libraries were generated using Illumina TruSeq DNA PCR-Free Library Preparation Kit (Illumina, USA) following manufacturer’s recommendations, and index codes were added. The library quality was assessed on the Qubit@ 2.0 Fluorometer (Thermo Scientific) and Agilent Bioanalyzer 2100 system. Finally, the library was sequenced on an Illumina NovaSeq platform and 250 bp paired-end reads were generated.

### Paired-end reads from the original DNA fragments are merged by using FLASH

1. Paired-end reads was assigned to each sample according to the unique barcodes. Sequences were analyzed using QIIME [17, 19].
2. Software package (Quantitative Insights into Microbial Ecology), and in-house Perlscripts were used to analyze alpha- (within samples) and beta- (among samples) diversity. First, reads were filtered by QIIME quality filters. Sequences with ≥ 97 % similarity were assigned to the same OTUs. A representative sequences for each OTU was picked, and used as the RDP classifier [20, 21].
3. In order to compute Alpha Diversity, we rarified the OTU table and calculated three metrics: Chao1 estimates the species abundance, Observed Species estimates the number of unique OTUs found in each sample, and the Shannon index. Rarefaction curves were generated based on these three metrics. QIIME calculates both weighted and unweighted unifrac, which are phylogenetic measures of beta diversity. To mine deeper data of microbial diversity of the differences between the samples, significance tests were conducted with some statistical analysis methods, including T-test, MetaStat, LEfSe, Anosim and MRPP [22, 23].

### Data analysis

#### Weight and body measure data analysis

Data were analyzed by Statistical Analysis System (SAS) (2013) as a randomized block design, considering the starch source as the main effect and the sampling time points as block. A mixed linear model (PROC MIXED ) was used to analyze body weight and body measurements differences between groups. The level of significance was *P* < 0.05, and the Ls means method is used for multiple comparison.

#### Alpha diversity and relative abundance analysis

Statistical analyses on the microbial dataset were performed in a R environment. Diversity and richness of gut flora for the three diet groups were displayed by the Shannon diversity and Chao1 richness indices. Student’s T-test, LEfSe, and Anosim were used to test the significance of differences in the species composition and community structure of the group samples. Tax4Fun was used to predict and analyze the microbial community in the samples.

## Results

### Growth performances

**Fig. 1:**
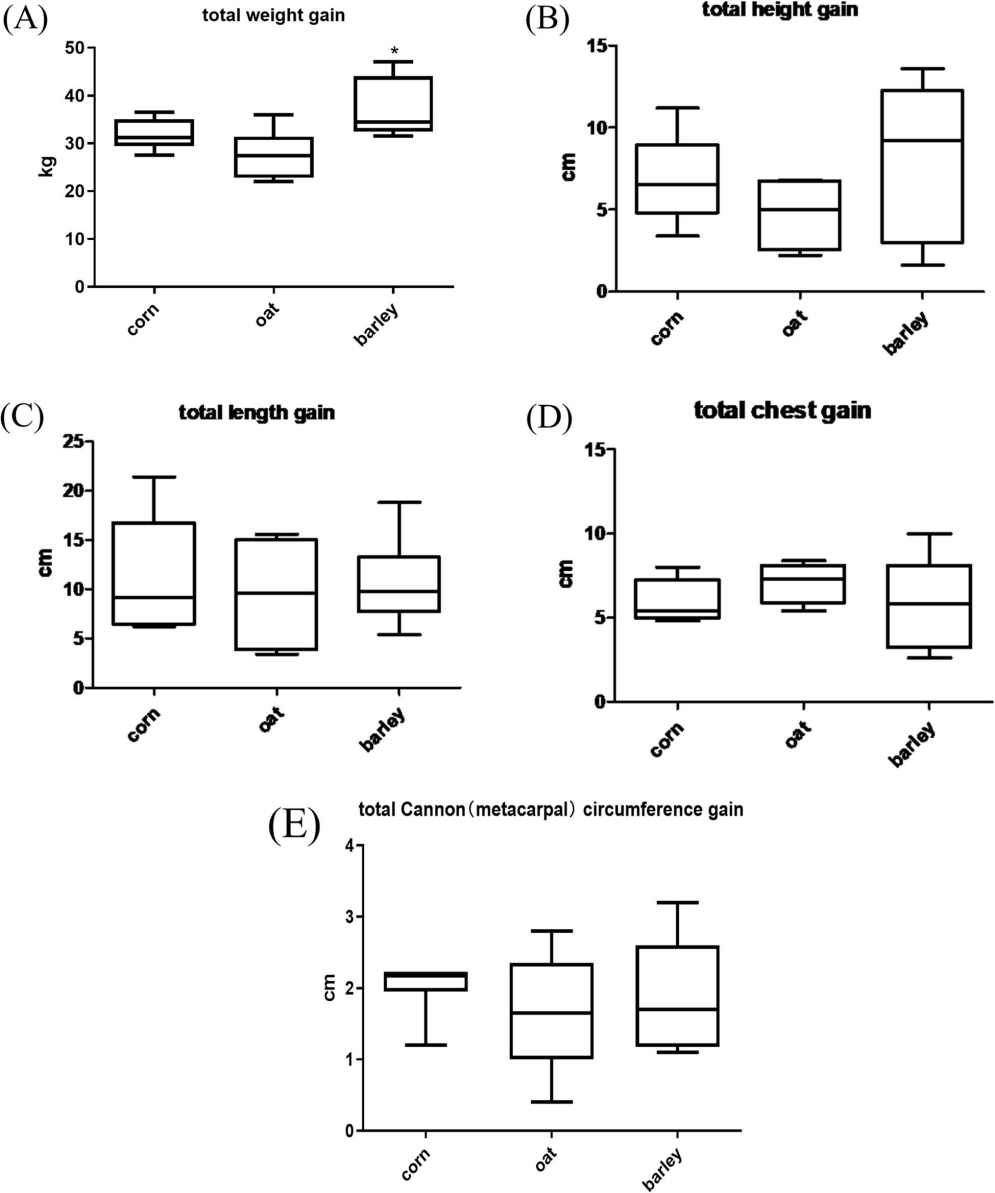
The effect of the source of grains on the total and average daily increases of body weight and body size of the foals. **a** Box and whisker plots showing the total weight gain of foals from different grain sources during the trial period. **b** A box-and-whisker plot showing the effect of different grain sources on the total body height of the foal during the trial period. **c** A box-and-whisker plot showing the effect of different grain sources on the total increase in foal body length during the trial period. **d** Box and whisker plots showing the effect of different grain sources on the total chest circumference of the foal during the trial period.(E) Box and whisker plots showing the increase in the total cannon circumference of foals from different grain sources during the trial period

Table 4 shows the effect of the grain source on the growth performance. Foals in the barley group were 5 kg heavier than those in the oats group, a difference that was significant (*P* = 0.0152), The barley group was also 3 kg heavier than the corn group, but this difference was not significant. The corn group was 2 kg heavier than the oats group, but this difference was not significant. As expected, all foals significantly (*P* < 0.01) grew over time. A significant treatment by time interaction could not be established. There were no significant differences amongst the diets in body height, body length, chest and cannon circumference of the foals (*P* > 0.05).The total increases in body weight and body size of the foals are shown in Fig. 1.The total weight gain of foals in barley group was significantly higher than that in oat group (*P* < 0.05).

**Table 4:** The effect of the source of grains on growth performances of foals

Item	Corn	Oat	Barley	SEM	*P* value		
					trt	date	trt×date
Body weight (kg)	127.33^ab^	125.05^b^	130.28^a^	1.5230	0.0509	< 0.0001	0.4238
Body height (cm)	117.61	116.82	117.44	1.1757	0.8707	0.0018	0.5372
Body length (cm)	114.46	112.57	114.06	1.4039	0.5803	< 0.0001	0.7843
Chest (cm)	110.99	109.81	112.06	1.4058	0.5061	< 0.0001	0.7567
Cannon circumference (cm)	14.56	14.20	14.46	0.1862	0.3355	< 0.0001	0.7979

Note: Different letters marked with ^abc^ on peer data indicate significant differences (*P*<0.05); the same letters marked with ^abc^ on peer data or no letters indicate non-significant differences (*P*>0.05)

### Alpha Diversity indices

Alpha diversity indices of the faecal bacteria [24] are shown in Table 5. There were no significant differences in PD whole_tree index, ACE index, or goods coverage between the diet groups. Significant differences were found for observed species index, Shannon index, Simpson index and Chao1 index between the diet groups. The observed species index was higher in the oats fed group than in the corn fed group (*P* < 0.05). The Shannon and Simpson indices were higher in the barley fed group than in the corn or oat fed groups (*P* < 0.05, *P* < 0.05, *P* < 0.05 and *P* < 0.05). The Chao1 index was higher in the oats group than either in the corn or barley group (*P* < 0.05 and *P* < 0.05).

**Table 5:** Alpha diversity indices of fecal microbiota

Items	Corn	Oat	Barley
Observed_species	1424.40 ± 52.68^b^	1561.17 ± 179.55^a^	1447.20 ± 60.76^ab^
PD_whole_tree	102.41 ± 18.68	120.05 ± 21.92	102.60 ± 31.88
Shannon	6.74 ± 0.26^b^	6.96 ± 0.61^b^	7.56 ± 0.51^a^
Simpson	0.96 ± 0.01^b^	0.95 ± 0.03^b^	0.98 ± 0.01^a^
Chao1	2291.39 ± 214.16^b^	2269.51 ± 163.55^a^	2133.51 ± 102.39^b^
ace	1729.13 ± 77.04	1767.14 ± 68.99	1727.12 ± 77.54
Goods_coverage	0.99 ± 0.00	0.99 ± 0.00	0.99 ± 0.00

Note: Different letters marked with ^abc^ on peer data indicate significant differences (*P*<0.05); the same letters marked with ^abc^ on peer data or no letters indicate non-significant differences (*P*>0.05)

### Venn graph based on operational taxonomic units

**Fig. 2:**
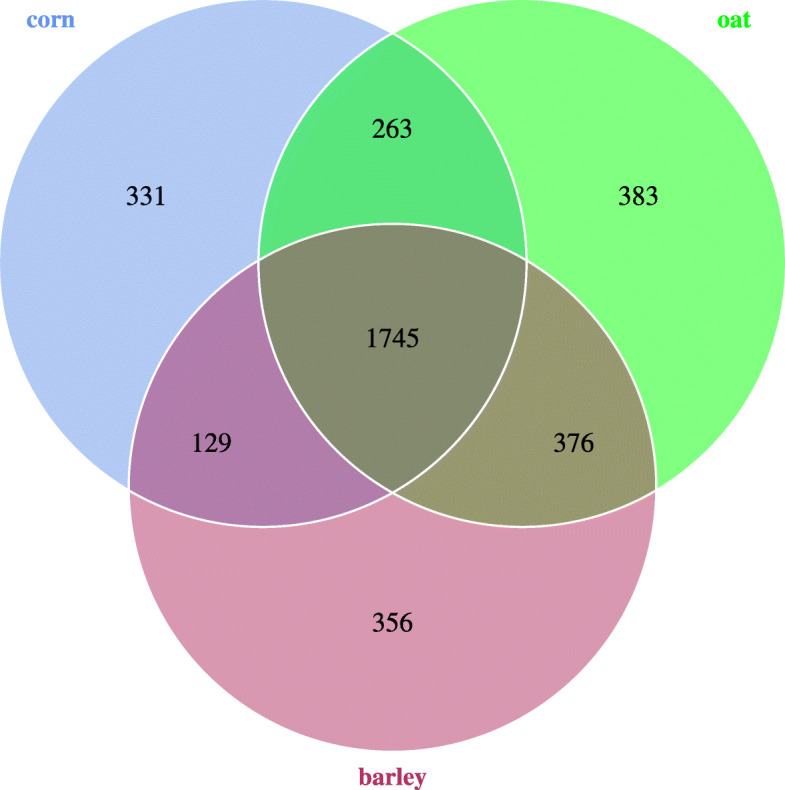
Venn graph based on OTU.Each circle in the figure represents a group of samples; the number of circles and the overlap of circles represents the number of common OTUs among all experimental groups; the number of unique OTUs among all experimental groups without overlap represents the number of unique OTUs among all experimental groups

The Venn diagram (Fig. 2) shows the logical relations between the OTUs numbers of the 3 sets, representing each one diet group. The number of shared OTUs was1745. The number of unique OTUs was 311 in the corn fed group, 383 in the oats fed group, and 356 in the barley fed group. The number of unique OTUs in the faeces of the oats fed group thus is higher than in the corn or barley fed foals.

### Relative abundance of faecal bacteria

**Fig. 3:**
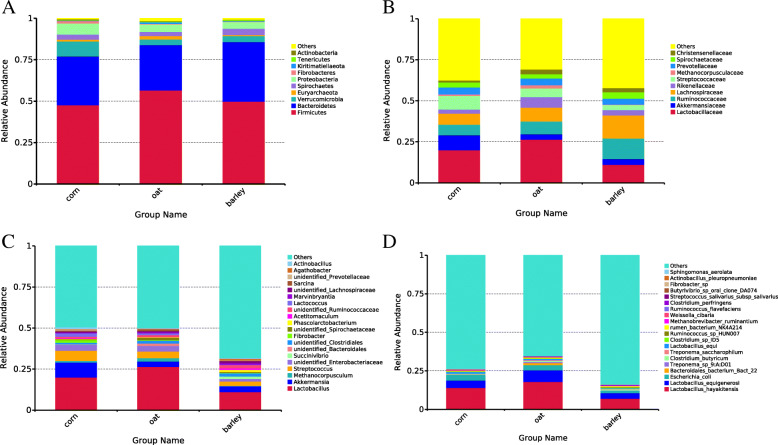
Relative abundance of faecal bacteria at the phylum (**a**), family (**b**), genus (**c**) and species (**d**) levels. **a** histogram of the relative abundances of the first 10 phylum levels of bacteria in foal feces, where “Other” refers to the sum of the relative abundances of all species at the eleventh and subsequent levels of the taxonomic level. **b** histogram of relative abundance of horizontal bacteria of the first 10 families in foal feces. **c** histogram of relative abundance of horizontal bacteria of the first 20 genera in foal feces. **d** histogram of the relative abundances of the first 20 levels of bacteria in foal feces

The relative abundance of faecal bacteria at the phylum (A), family (B), genus (C) and species (D) levels are shown in Fig. 3. The top ten bacteria found in the faecal samples at the phylum level were Firmicutes, Bacteroidetes, Verrucomicrobia, Euryarchaeota, Spirochaetes, Proteobacteria, Fibrobacteres, Kiritimatiellaeota, Tenericutes, and Actinobacteria. The majority of bacteria found in all faecal samples throughout the trial belonged to the phyla Firmicutes (48 % in corn fed foals, 57 % in the oats fed foals and 50 % in the barley fed foals) and Bacteroidetes (29 % in the corn group, 27 % in the oats group and 36 % in the barley group). Tenericutes was significantly more abundant in samples from oats fed foals compared to the corn fed foals (*P* = 0.027). Actinobacteria was significantly more abundant in the corn group than in the barley group (*P* = 0.028).

At the family levels, the 10 top bacteria were Lactobacillaceae, Akkermansiaceae, Ruminococcaceae, Lachnospiraceae, Rikenellaceae, Streptococcaceae, Methanocorpusculaceae, Prevotellaceae, Spirochaetaceae and Christensenellaceae. Regarding relative abundance, the Rikenellaceae were significantly more abundant in the oats fed group compared to the corn fed group (*P* = 0.039). The Streptococcaceae were more abundant in the corn fed group compared to the oat group and barley fed groups (*P* = 0.001 and 0.010). The Lactobacillaceae were more abundant both in the corn and oats group compared to the barley group (*P* = 0.039 and 0.001). The Lachnospiraceae were more abundant in the barley fed group compared to the corn fed group (*P* = 0.023).

At the genus levels, there were significant differences in the relative abundance of the top 20 bacteria. *Streptococcus* was more abundant in the corn fed group compared to the oat and barley fed groups (*P* = 0.012 and 0.015). *Lactococcus* was more abundant in the corn fed group compared to the oats and barley fed groups (*P* = 0.021 and 0.002), and the barley fed group compared to the oats fed group (*P* = 0.006). *Actinobacillus* was more abundant in the corn fed group compared to the oat and barley fed groups (*P* = 0.002 and 0.013). *Lactobacillus* was more abundant both in the corn and oat fed groups compared to the barley fed group (*P* = 0.049 and 0.010). *Agathobacter* was more abundant in the barley fed group compared to the oat fed group (*P* = 0.041).

At the species levels, there were 20 top bacteria identified : *Lactobacillus hayakitensis*, *Lactobacillus equigenerosi*, *Escherichia coli*, *Bacteroidales bacterium Bact 22*, *Treponema sp 9:A:D01*, *Clostridium butyricum*, *Treponema saccharophilum*, *Lactobacillus equi*, *Clostridium sp ID5*, *Ruminococcus sp HUN007*, *rumen bacterium NK4A214*, *Methanobrevibacter ruminantium*, *Weissella cibaria*, *Ruminococcus flavefaciens*, *Clostridium perfringens*, *Streptococcus salivarius subsp salivarius*, *Butyrivibrio sp oral clone DA074*, *Fibrobacter sp*, *Actinobacillus pleuropneumoniae*, and *Sphingomonas aerolata*.

### Linear discriminant analysis (LDA) effect size (LEfSe)

**Fig. 4:**
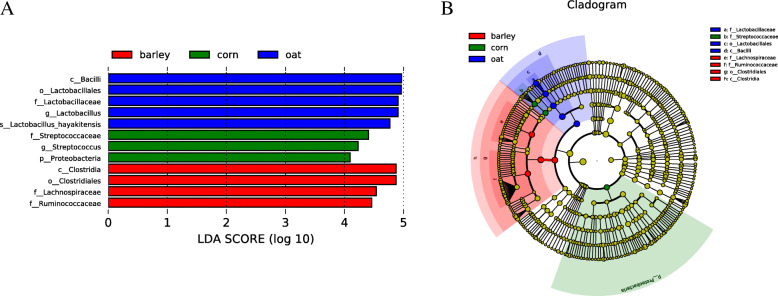
LDA Effect Size of statistically different biomarkers in fecal.Non-parametric factorial Kruskal-Wallis (KW) Sum-Ranktest (non-parametric Kruskal-Wallis and ranktest) was used to detect the species with significant difference in bacterial abundance in the feces of foals from different grain sources, and then the difference between groups was determined by group Wilcoxon rank sum test.Finally, linear discriminant analysis (LDA) was used to reduce and evaluate the impact size of significantly different species (i.e., LDA Score). **a** Histogram of LDA value distribution, showing the biomarkers with statistical differences between groups with LDA Score greater than the set value of 4.The length of the bar chart represents the influence size of different species (i.e., LDA Score). The blue is the species with significant abundance difference in the oat group, the green is the species with significant abundance difference in the maize group, and the red barley group. **b** Evolutionary branching diagram under LEFSE analysis.In the figure, the circles radiating from the inside to the outside represent taxonomic levels from phylum to genus (or species).Each small circle at different taxonomic levels represents a taxon at that level, and the diameter of the small circle is proportional to the relative abundance.Coloring principle: no significant difference of species uniform color is yellow, differences between species Biomarker follow group staining, red nodes play an important role in the barley group of microbial groups, said green nodes play an important role in corn group of microbial groups, said the blue nodes play an important role in the oat group of microbial groups.)

LEfSe [22] was used to find high-dimensional biomarkers for comparison between groups. Figure 4 shows the characteristics of different abundance and associated categories of the results. The histogram of LDA value distribution (Fig. 4a) shows that the statistically different biomarkers bacteria were Bacilli, Lactobacillales, Lactobacillaceae, *Lactobacillus* and *Lactobacillus hayakitensis* in the oats fed group; the Streptococcaceae, *Streptococcus* and Proteobacteria in the corn fed group; and the Clostridia, Clostridiales, Lachnospiraceae and Ruminococcaceae in the barley fed group. The evolutionary branch (phylogenetic distribution) and the abundance comparison of biomarkers showed eight bacteria: Lactobacillaceae, Streptococcaceae, Lactobacillales, Bacilli, Lachnospiraceae, Ruminococcaceae, Clostridiales and Clostridia in all groups, respectively (Fig. 4b).

### Prediction of function prediction of faecal bacteria

**Fig. 5:**
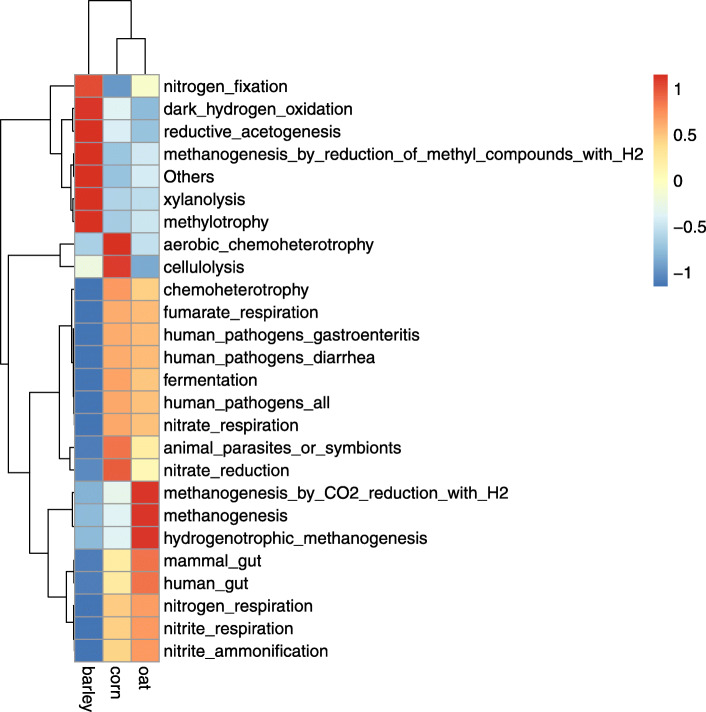
Tax4Fun function annotation clustering heat map.According to the functional annotation and abundance information of the samples in the data, the top 25 functions in the list of abundance and the abundance information of each group of samples were selected to draw a heat map, and the clustering was conducted from the level of functional differences.Functional notes are on the right side of the heat map, and test groups are on the lower side of the heat map.In the heat map, the red color represents the higher relative abundance of functions, and the relative abundance increases with the deepening of the color.Blue represents the higher the relative abundance of the function, and with the deepening of the color, the relative abundance decreases

In our study, Tax4Fun [25] was used for accurate predicting of the function of feaces samples. The Tax4Fun produced a functional annotation clustering heat map of foal faecal samples as shown in Fig. 5. We detected functional information of 26 species of bacteria in foal faecal samples. In the barley fed group, 7 species were significantly increased and showed the following functional information: nitrogen fixation, ark hydrogen oxidation, others, reductive acetogenesis, xylanolysis, methanogenesis by reduction of methyl compounds with H_2_, methylotrophy and aerobic chemoheterotrophy. In the corn fed group, we detected a significant increase of four species with functional information on aerobic chemoheterotrophy, cellulolytic, parasites or symbionts and nitrate reduction. In the oats fed group, three species displaying functional information methanogenesis by CO_2_ reduction with H_2_, methanogenesis, and hydrogenotrophic methanogenesis were significantly increased.

## Discussion

The aim of this study was to show that sources of starch from steam-flacked barley, corn and oats in the diet of foals cause differences in growth. The rationale for this assumption is that in plants amylose and amylopectin in the starch granules form dense hydrogen bonds to various degrees that hinder the action of digestive enzymes, and thereby reduce starch pre-ceacal fermentation [26]. The higher the ratio of amylose to amylopectin is, the lower is the digestibility of starch [7]. Steam treatment of cereals is a common method to improve starch digestibility and is considered superior to crushing [27–29] reported improvement of average daily gain and feed efficiency of calves fed steam-flaked corn grain. Therefore, we used steam-flaked oats, corn and barley in our study.

We showed that 2 g starch (DM) from barley /kg BW daily added to a routine standard diet stimulated body weight gain of foals significantly more than did additional starch from corn and oats. Although Potter et al. [30] suggested that the upper limit of starch digestion in the equine small intestine is between 3.5 and 4 g starch/kg body weight per feeding we decided in respect to the young age of the horses to 2 g/kg body weight per day in order to limit starch bypass to the large intestine [6, 31, 32]. In this study the total daily starch ingestion in the 3 diet groups appeared on the safe side and did not result in digestive abnormalities.

Corn, oats and barley contain similar amounts of starch but due to their different amylose-amylopectin ratios and the different size of starch granules, digestibility for horses is different [7, 33]. The starch that is not digested by enzymes in the horse’s small intestine is transported to the hind gut [34–36] and is fermented by the residing microbial population which includes starch-degrading bacteria. Their action may cause fundamental changes in the structure of the hind gut flora and result in increasing concentrations of volatile fatty acids and lactic acid [37]. In our study feeding oats significantly increased the numbers of observed species and the (Chao l index), whereas feeding barley only increased bacterial diversity (Shannon index) significantly. This implicates that varying types of dietary cereal starch could change the structure and diversity of bacterial population in foal’s hind gut. In case of feeding oats there was a relative increase in abundance of Tenericutes, Rikenellaceae and Lactobacillus in the faecal samples.

The results of our study cannot be compared in detail with other studies, but our finding in foals showed that similar effects were seen in adult horses in the trail by Harlow et al. [6] as far as concerns oats and corn feeding. The number of amylolytic bacteria increase by feeding corn and wheat, but not by oats [6]. whereas the decrease in cellulolytic bacteria were larger in horses fed corn and wheat middlings. Furthermore, the predominant amylolytic isolates from corn fed and wheat fed horses was Enterococcus faecalis, but other species were found in oat fed horses.

In our study, the LEfSe showed that fibrolytic Ruminococcaceae amounts were significantly different in faecal matter from barley fed foals than for oats and corn fed foals which is corroborated by findings of Bulmer et al. [38] who showed that even a small addition of starch to the diet was enough to reduce this bacterial Ruminococcaceae population. We found that the relative abundance of Ruminococcaceae in all 3 diet groups were *Ruminococcus_sp_HUN007* and *Ruminococcus flavefaciens*.Relative abundances in Lactobacillales, Lactobacillaceae, *Lactobacillus* and *Lactobacillus hayakitensis* were greater the oat fed group compared to the corn and barley fed group. An increase in lactic-acid producing bacteria has also been reported to be coupled with a corresponding decrease in fibrolytic bacterial abundance by Daly et al. [4].

The results of functional prediction showed that long-term feeding of three different cereals may have varying effects on digestive physiology of foals. Bulmer et al. [38] showed that the dietary change could result in changed behavior and faecal microbiota. In our study, we didn’t observe the abnormal behavior.

In our study, 16 S RNA sequencing technology was used to determine the bacterial community in foal feces, and the bacterial classification and composition at phylum, family, genus and species levels were analyzed from the bacterial classification level. The results showed that starch intake affected coliform groups and structure. However, this study did not further study the enzymatic hydrolysis of starch from different sources and its influence on fiber degrading bacteria, and did not further clarify the influence of starch intake on the species and number of lactic acid bacteria and fiber degrading bacteria through bacterial culture technology. Next, we will examine the effects of starch sources on foal hindgut ecosystem and health by studying starch intake, amylose to amylopectin ratio, digestibility, determination of ph changes in faeces, lactic acid production and volatile fatty acid production.

## Conclusions

The current study showed differences in the faecal microbiota of foals fed either corn, oat or barley, and also differences in the overall growth of the foals. Different types of grain have different impact on faecal microbiota, which is mainly caused by different starch digestibility. Further investigation is required to look at the potential impact of changes in the microbiota on the functional impact on foals when fed grains.

## Data Availability

All data generated or analysed during this study are included in this published article.
